# Towards Machine Learning-Based Detection of Running-Induced Fatigue in Real-World Scenarios: Evaluation of IMU Sensor Configurations to Reduce Intrusiveness

**DOI:** 10.3390/s21103451

**Published:** 2021-05-15

**Authors:** Luca Marotta, Jaap H. Buurke, Bert-Jan F. van Beijnum, Jasper Reenalda

**Affiliations:** 1 Roessingh Research and Development, 7522 AH Enschede, The Netherlands; j.buurke@rrd.nl (J.H.B.); j.reenalda@rrd.nl (J.R.); 2 Department of Biomedical Signals and Systems, Faculty of Electrical Engineering, Mathematics and Computer Science (EEMCS), University of Twente, 7522 NB Enschede, The Netherlands; b.j.f.vanbeijnum@utwente.nl

**Keywords:** fatigue estimation, biomechanics, IMU, machine learning, human movement, running

## Abstract

Physical fatigue is a recurrent problem in running that negatively affects performance and leads to an increased risk of being injured. Identification and management of fatigue helps reducing such negative effects, but is presently commonly based on subjective fatigue measurements. Inertial sensors can record movement data continuously, allowing recording for long durations and extensive amounts of data. Here we aimed to assess if inertial measurement units (IMUs) can be used to distinguish between fatigue levels during an outdoor run with a machine learning classification algorithm trained on IMU-derived biomechanical features, and what is the optimal configuration to do so. Eight runners ran 13 laps of 400 m on an athletic track at a constant speed with 8 IMUs attached to their body (feet, tibias, thighs, pelvis, and sternum). Three segments were extracted from the run: laps 2–4 (no fatigue condition, Rating of Perceived Exertion (RPE) = 6.0 ± 0.0); laps 8–10 (mild fatigue condition, RPE = 11.7 ± 2.0); laps 11–13 (heavy fatigue condition, RPE = 14.2 ± 3.0), run directly after a fatiguing protocol (progressive increase of speed until RPE ≥ 16) that followed lap 10. A random forest classification algorithm was trained with selected features from the 400 m moving average of the IMU-derived accelerations, angular velocities, and joint angles. A leave-one-subject-out cross validation was performed to assess the optimal combination of IMU locations to detect fatigue and selected sensor configurations were considered. The left tibia was the most recurrent sensor location, resulting in accuracies ranging between 0.761 (single left tibia location) and 0.905 (all IMU locations). These findings contribute toward a balanced choice between higher accuracy and lower intrusiveness in the development of IMU-based fatigue detection devices in running.

## 1. Introduction

Running is an increasingly popular sport, with multiple health benefits. Fifty million Europeans engage in running according to a recent estimate [1]. Health benefits can be psychological, such as a sense of accomplishment [2], or physical, such as a decreased chance of developing chronic diseases and higher longevity [3]. However, running comes with associated risks, in particular pain and injuries [4]. To maximize health benefits and minimize chances of being injured, load should be carefully managed. For instance, overloading and training stress are associated with increased injury risk [5,6]. Accurate, continuous detection of fatigue during a high intensity or long duration running activity can be used to provide feedback to runners in order to avoid excessive training stress and overloading which can lead to lower limb injuries.

Fatigue is a multi-factorial phenomenon. A model developed by Kluger et al. [7] divides fatigue in two distinct components that have the capacity to influence each other: perception of fatigue, caused by homeostatic and psychological factors, and performance fatigability, which is influenced by central and peripheral factors. During running, the human body undergoes shocks due to impacts with the ground. Performance fatigability can be assessed e.g., by means of changes in biomechanical quantities that are related to coping with such shocks. However, fatigue identification and management are commonly based solely on subjective estimates of fatigue that measure perception of fatigue. Subjective estimates of fatigue are very easy to use in practice, but they lack any assessment of performance fatigability.

Inertial measurement units (IMUs) are non-intrusive sensors widely adopted to measure biomechanical changes in human movement. IMUs can record biomechanical parameters continuously, which can show changes due to physical fatigue [8,9]. Extensive research has been performed to detect biomechanical changes due to fatigue in running. Hip flexion at initial contact was found to decrease between the start and the end of a fatiguing run [8,10]. Maximum knee flexion angle can decrease [8] or increase [11] with fatigue depending on different running settings and subject characteristics. Maas et al. [12] showed that running experience could influence knee flexion, among other biomechanical parameters. Peak tibial (PTA) and peak sacral (PSA) accelerations are also recurring parameters studied in association with fatigue. Reenalda et al. [13] and Schutte et al. [14] found an increase in PTA due to fatigue, while Ruder et al. [15] found a decrease. Reenalda et al. further investigated shock attenuation between the tibia and the pelvis, finding an increase due to fatigue although both PTA and PSA increased as a consequence of fatigue [13]. Assessment of asymmetry in ankle, knee, and hip kinematics between a rested and fatigued state in running resulted in internal rotation of the knee showing the largest increase in asymmetry with fatigue [16]. Although significant changes in biomechanics have been repeatedly found when measuring running mechanics with IMUs, it is not clear yet whether these changes are sufficient to reliably detect fatigue over time in real-world applications.

While fatigue detection in running has been based on non-automized detection of changes in biomechanical parameters, machine learning algorithms could have the benefit of rapid and easy application to identify fatigue. Machine learning algorithms could use as an input well-established biomechanical variables, as well as a wide range of statistical variables. Translation of biomechanical changes due to fatigue into machine learning fatigue detection algorithms has been performed in other fields. A clear example of such practice is in the area of industry work. Feeding a wide range of biomechanical parameters into a support vector machine classification algorithm led to a fatigue detection accuracy of 90% in working tasks [17]. Yet, few studies have focused on the detection of a fatigue condition in sports and running, especially in out-of-the-lab environments. Gholami et al. used machine learning techniques to detect the perceived exertion of runners on a treadmill using textile wearable sensors and assessed the importance of each sensor location, with the hip contributing more than the knee and the ankle to the final coefficient of determination of 0.96 [18]. Buckley et al. located IMUs at the shanks and lumbar spine and compared three different locations and various machine learning classifiers to detect fatigue in outdoor running, obtaining a 75% accuracy with a single IMU placed at the lumbar spine [19]. While minimal sensor setups present the unequivocal advantage of being easy to wear, they might be missing substantial biomechanical information to improve fatigue detection accuracy.

Here we aimed to assess the optimal combination of IMU locations at the lower limbs and trunk to detect fatigue levels in an outdoor run with a machine learning classification algorithm. We segmented IMU data into gait cycles and extracted biomechanical and statistical features, labeling data points with fatigue levels identified by means of subjective assessment of fatigue and heart rate (HR). IMU combinations of interest were selected and their fatigue detection performance was compared. It was hypothesized that larger biomechanical changes reported in the literature such as peak tibial accelerations would reflect in the combinations of sensor with higher fatigue detection accuracy. However, we expected statistical features derived from biomechanical quantities to also have a positive impact in the performance of the classifier. Findings of this study aim to assist translating current state-of-the-art knowledge of the biomechanical changes due to fatigue in running into detection of fatigue in real-world scenarios.

## 2. Materials and Methods

**Figure 1:**
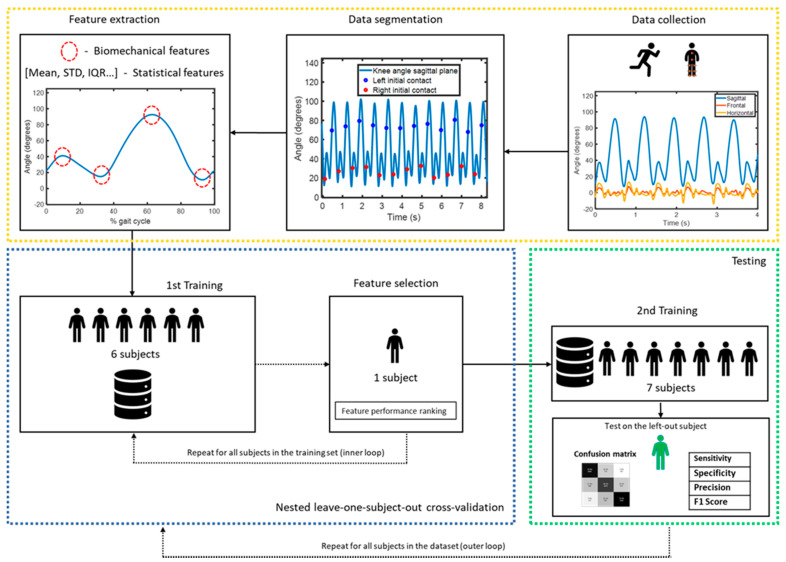
Machine learning fatigue detection algorithm workflow.

The machine learning-based method that we implemented to detect fatigue in this study is summarized in Figure 1. Our workflow consisted of three main stages: data collection and processing (highlighted in yellow), development of the fatigue detection classifier (highlighted in blue) and performance evaluation of the classifier (highlighted in green). Each step in the workflow will be described in more detail in this section.

### 2.1. Experimental Design

Eight healthy runners were recruited (3 males 5 females, 24.3 ± 1.0 years, 174.8 ± 9.5 cm, 71.1 ± 8.8 kg, Table 1). Inclusion criteria consisted of the absence of major injuries in the previous year and having run at least 10 km per week in the previous six months. The experimental protocol was approved by the Medical Ethical Review Committee ‘CMO Arnhem-Nijmegen’ and all participants signed an informed consent form prior to participation.

Subjects underwent a fatiguing protocol consisting of three distinct consecutive runs:

1. The first run consisted of a 4000 m run (ten laps of the athletic track) at a constant speed, determined as the average speed of the subject during the best performance in the previous year on a 5 to 10 km race;
2. The second run was performed according to a fatiguing protocol. The speed in this fatiguing protocol started at the same level of the first run and increased progressively by 0.2 km/h every 100 m. Perceived fatigue was assessed by means of a Borg Rating of Perceived Exertion (RPE) Scale (min-max score 6–20) [20], asked to the runner every 100 m. The fatiguing protocol was terminated once the RPE was equal to 16 (RPE between ‘hard’ and ‘very hard’) or higher, or, if such requirement was not met, after 1200 m;
3. The third run consisted of a 1200 m run (three laps of the athletic track), in which speed was kept constant and equal to the first 4000 m run.

Speed was controlled throughout the whole experimental protocol using a cyclist, proceeding at constant speed approximately 2 m in front of the runner. Except for the fatiguing protocol, half of each run was performed in clockwise direction and the other half in counterclockwise direction, in a randomized fashion to eliminate the effect of running direction on the biomechanics of the left and right leg.

### 2.2. Measurement Setup

**Figure 2:**
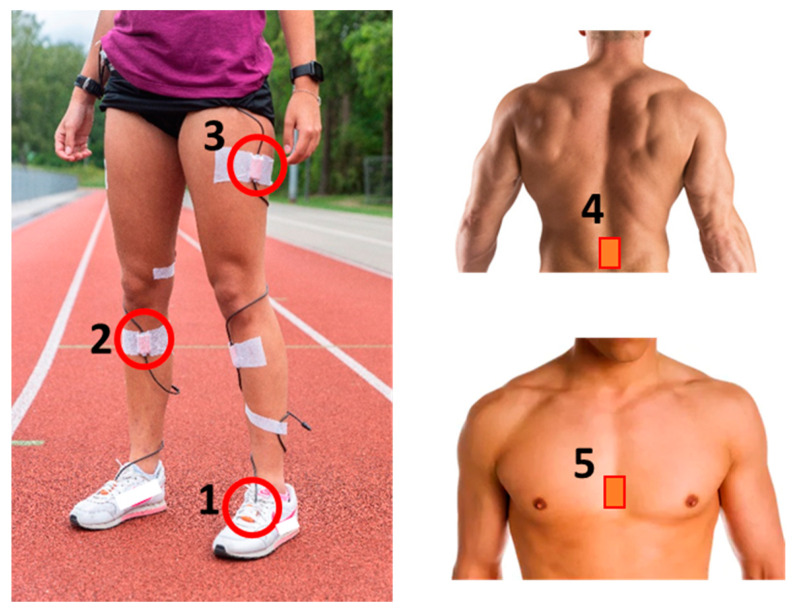
Measurement setup. IMUs are placed at both feet (**1**, highlighted left foot), both tibias (**2**, highlighted right tibia), both thighs (**3**, highlighted left thigh), pelvis (**4**) and sternum (**5**).

Xsens MTx IMUs (Xsens Technologies B. V., Enschede, The Netherlands) were attached with kinesiotape to eight body locations of the runner throughout the whole running experiment: left and right foot, left and right tibia, left and right thigh, pelvis and sternum (Figure 2). Double-sided tape was also attached between the IMU and the skin to limit skin artefacts. 3D accelerometer range of the IMUs is 16 g, 3D angular velocity range is 1200 °/s, sampling frequency is 240 Hz. Running speed and HR were recorded simultaneously using a GPS watch (Garmin Forerunner 210, Garmin, Wichita, KS, USA). The bicycle speed was measured with a bicycle computer (Sigma BC 16.16 STS, Sigma, Neustadt, Germany) and shown in real time to the cyclist on a display.

### 2.3. Data Acquisition

MVN Analyze (v2019.2.1, Xsens Technologies B. V., Enschede, The Netherlands) was used for data acquisition. A Kalman filter fusing accelerometers, gyroscopes and magnetometers data were used to estimate joint angles (left and right ankle, left and right knee, left and right hip) and segmental accelerations and angular velocities (left and right foot, left and right tibia, left and right thigh, pelvis and sternum) together with a biomechanical model [21].

### 2.4. Data Analysis

**Figure 3:**
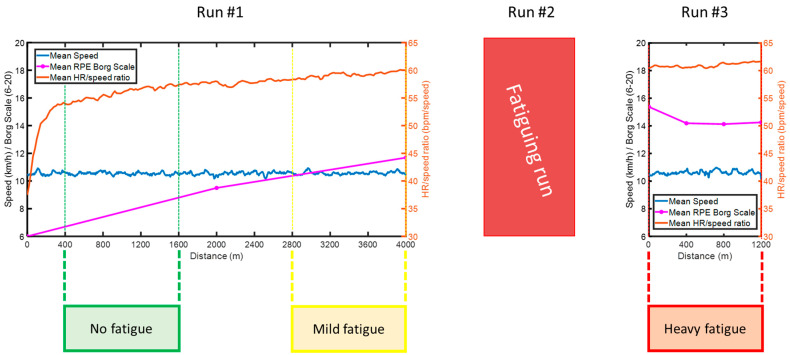
Mean speed (km/h), RPE Borg Scale (6–20) and HR/speed ratio (bpm/speed) for the runs performed in this study. The first run lasted 4000 m and was performed at a speed level controlled by a cyclist; the second (fatiguing) run was performed at increasingly higher speed and lasted between 400 m and 1200 m, until a RPE of 16 or higher was reached; the third run lasted 1200 m and was performed at a speed level controlled by a cyclist and equal to the first run. The three identified fatigue conditions are highlighted in green (400–1600 m), yellow (2800–4000 m) and red (0–1200 m post fatiguing run).

Three segments were extracted from the runs: laps 2–4 from the first run, identified as no fatigue condition (RPE = 6.0 ± 0.0); laps 8–10 from the first run, identified as mild fatigue condition (RPE = 11.7 ± 2.0); laps 1–3 from the third run, identified as heavy fatigue condition (RPE = 14.2 ± 3.0). The second run served only as a fatiguing protocol and differed in length per subject, therefore was not included in the analysis. As per Figure 3, mean RPEs increased throughout the first run, and decreased during the last run after the fatiguing protocol (although considerably higher than the RPEs pre-fatiguing run), while HR kept increasing throughout the runs.

### 2.5. Data Processing

MATLAB R2019a (The MathWorks Inc., Natick, MA, USA) was used for data processing. Running gait segmentation was performed based on the pelvis velocity. First, the start and the end of each run were detected with the zero-crossing of the pelvis velocity in the sagittal plane. Then, downward peaks in pelvis velocity were calculated by means of a peak detection algorithm [22]. Left and right initial contact timepoints were determined based on the right knee angle. Joint angles, segmental acceleration magnitudes ($a=\sqrt{a_{x}^{2}+a_{y}^{2}+a_{z}^{2}\;}$) and angular velocities in all three dimensions were cut into gait cycles starting at each initial contact and normalized at 150 data points.

### 2.6. Feature Extraction and Processing

A total of 157 features were extracted from each gait cycle for all subjects. Features were extracted from eight body segments (Table 2: left and right foot, left and right tibia, left and right thigh, pelvis, and sternum) and six joint angles (left and right ankle, left and right knee, left and right hip). The 157 features consist of 43 biomechanical features (based on reported changes in biomechanics due to fatigue in running [8,9,10,11,12,13,14,15,16]), 110 statistical features and four spatiotemporal features, computed as follows:

- The 43 biomechanical features were extracted from body segments and joint angles. 18 features were extracted from the body segments: eight peak segmental acceleration magnitudes, one per segment; eight peak pitch angular velocities (in the sagittal plane), one per segment; two shock attenuation features (defined as in [13]), between the left/right tibia and the pelvis. Five features were extracted from the lower limb joints: 22 joint angles maxima and minima; three symmetry features were computed between the left and right joint angle at each joint level (ankles, knees, hips).
- The 110 statistical features were extracted from joint angles, segmental accelerations and angular velocities. Mean, standard deviation (STD), inter-quartile range (IQR), skewness and kurtosis were selected in order to assess gait variability.
- The four spatiotemporal features consisted of stride time and stride length, extracted from the left and the right gait cycle.

A moving average over one full lap was applied to all features. The sliding window length of 400 m (one full lap on the athletic track) was chosen in order to minimize the effects of running direction on the biomechanical features. Z-score normalization of features was performed for each subject since speed has an effect on biomechanical parameters, in particular tibial acceleration [23].

### 2.7. Dataset Composition

**Figure 4:**
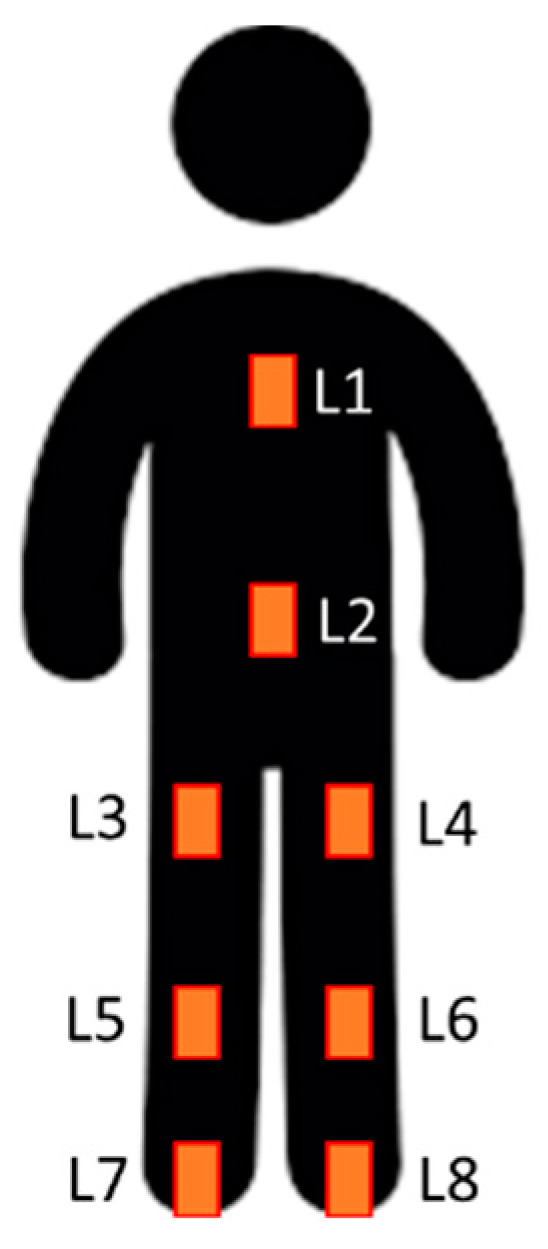
IMU location codes: **L1**, sternum; **L2**, pelvis; **L3**, right thigh; **L4**, left thigh; **L5**, right tibia; **L6**, left tibia; **L7**, right foot; **L8**, left foot.

The number of possible combinations for eight IMU locations is 255 (Table 3). While the sum of 1-IMU combinations is only eight, some of the multiple IMUs configurations result in a high number of possible combinations that would be too complex to analyze in this study. Among the 255 combinations, some carry more value than others. Each of the eight IMU locations was identified with a code (Figure 4). We divided IMU combinations of interest in four categories (Table 4): minimally intrusive, including only one IMU; quasi-minimally intrusive, including two adjacent IMUs; 3+ IMUs, including three or four IMUs; whole body, including all eight IMUs.

### 2.8. Machine Learning Pipeline

Datapoints were divided in three fatigue classes (Section 2.4): those derived from laps 2–4 were labeled as ‘*no fatigue*’ condition, while those derived from laps 8–10 were labeled as ‘mild fatigue’ and those derived from laps 11–13 (post fatiguing protocol) were labeled as ‘heavy fatigue’. Feature vectors were then used to train a random forest classification algorithm with 100 ensemble trees. A nested leave-one-subject-out cross validation was performed [24]. In the inner loop, the best features were learned for each IMU configuration by permuting out-of-bag observations among the trees, each time excluding one subject from the training set as well as the test subject (Figure 1). Then, the random forest classifier was trained on the selected feature vectors from all subjects in the training set. The 12 best features were selected for all IMU configurations consisting of two or more sensors, allowing a comparison with minimally intrusive configurations (each consisting of 12 features). Training and test sets were then re-partitioned, each time with the test set consisting of datapoints from a different subject and the training set consisting of datapoints from all remaining subjects (outer loop). Confusion matrices, accuracy, sensitivity, specificity, precision and F1 score were then used to compare performance of the different IMU configurations. All performance metrics were computed as an average from all eight left-out-subjects.

## 3. Results

**Figure 5:**
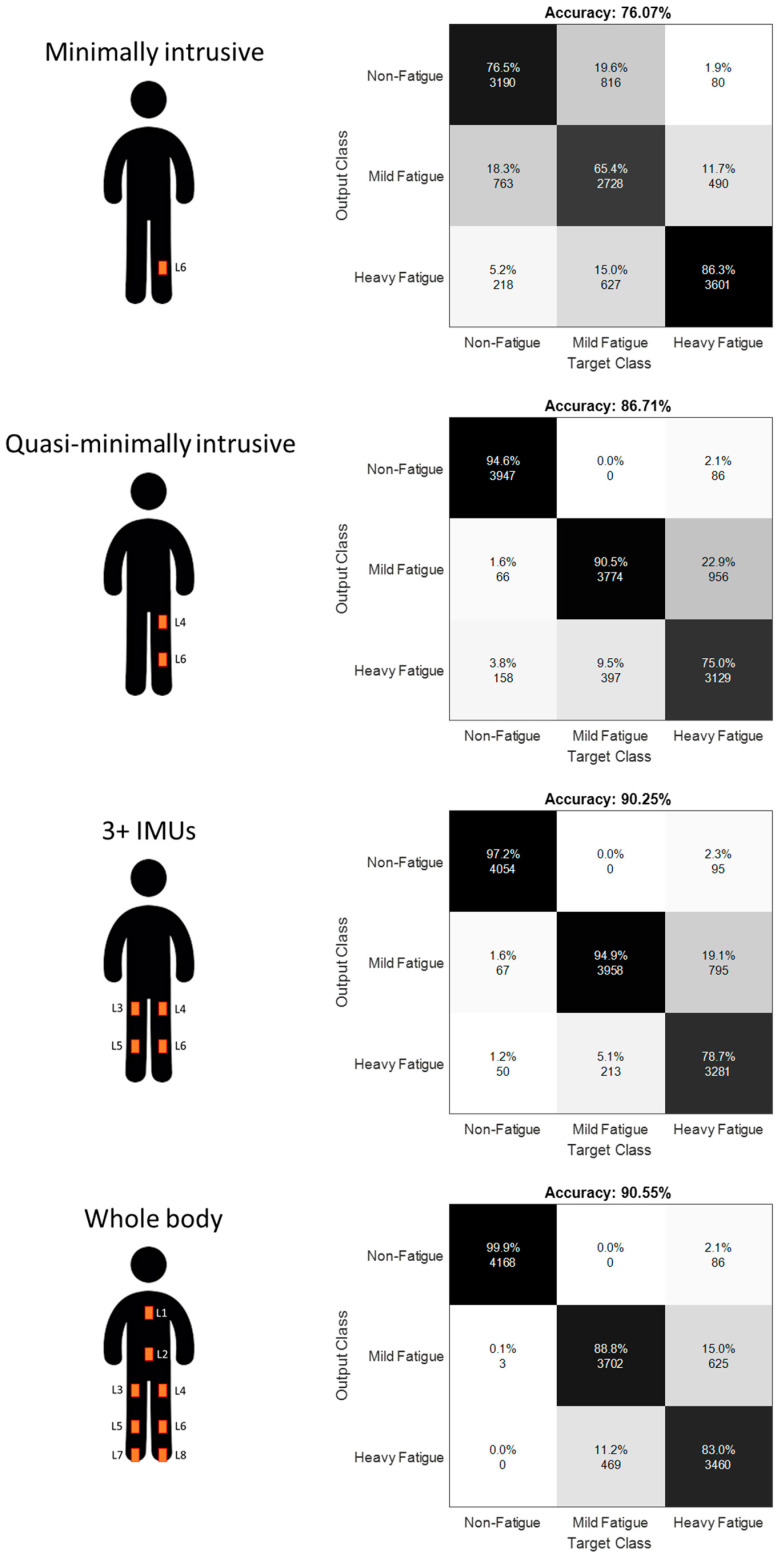
Aggregate confusion matrices for the IMU configurations resulting in higher accuracy in each category.

Table 5 presents the performance of our random forest classification algorithm in detection of the three fatigue levels for the sensor configuration that resulted in highest accuracy from each of the four IMU-setup configuration categories. For the minimally intrusive category, best configuration consists of one IMU placed on the left tibia (Accuracy = 0.761 ± 0.220). For the quasi-minimally intrusive category, best configuration consists of two IMUs placed on the left tibia and the left thigh (Accuracy = 0.867 ± 0.112). For the 3+ IMUs category, best configuration is represented by four IMUs on the left and right tibias and left and right thighs (Accuracy = 0.903 ± 0.085). Whole body configuration of eight IMUs resulted in an accuracy of 0.905 ± 0.081. Confusion matrices with aggregate classification accuracy for all datapoints from all subjects are shown in Figure 5.

**Figure A1:**
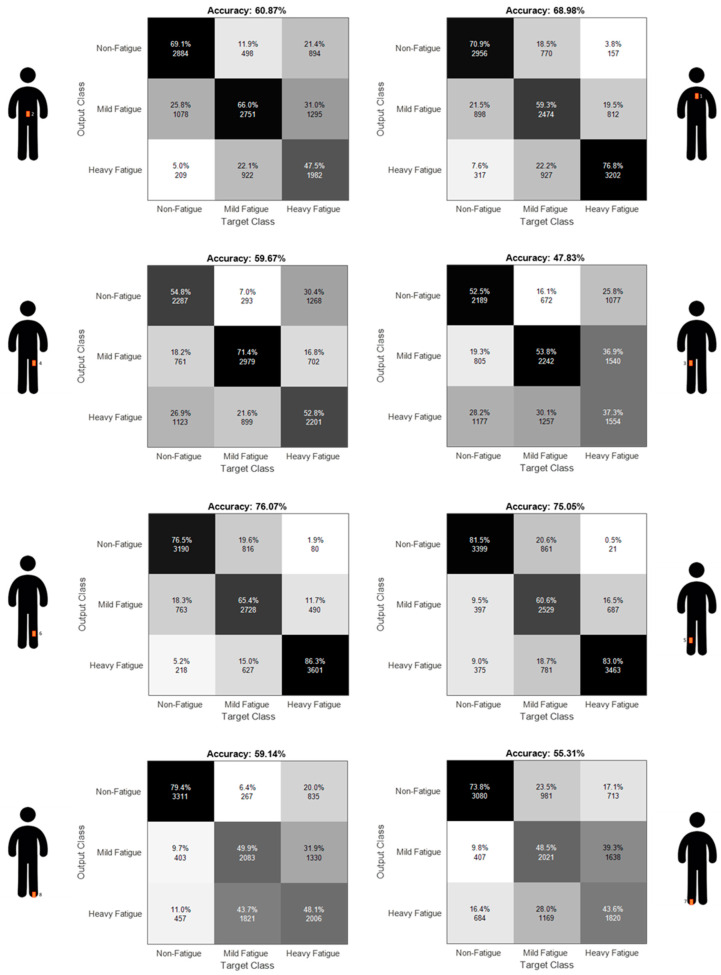
Aggregate confusion matrices with all subjects, minimally intrusive IMU configurations.

**Figure A2:**
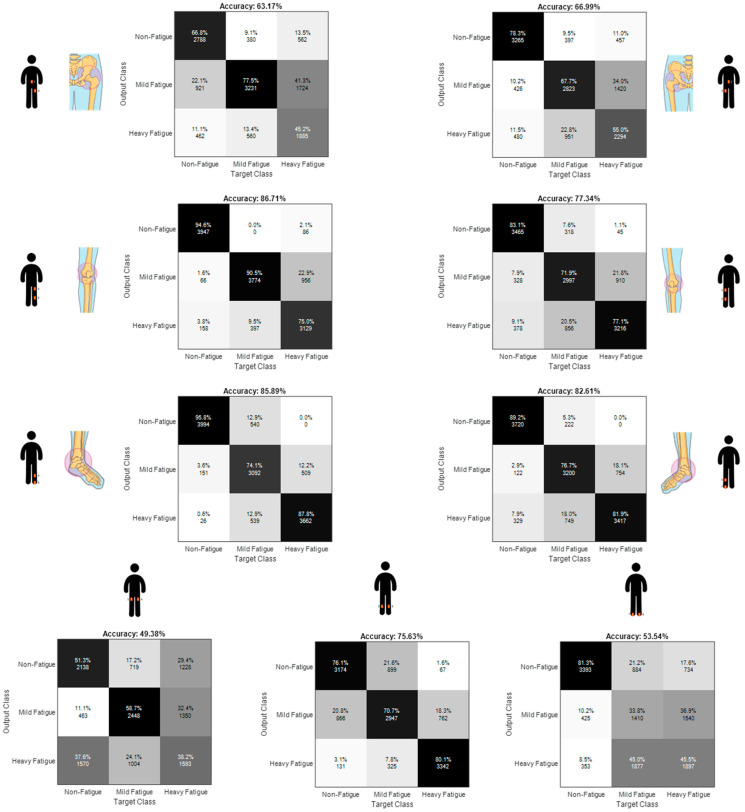
Aggregate confusion matrices with all subjects, quasi-minimally intrusive IMU configurations. Joints present in each configurations are shown.

**Figure A3:**
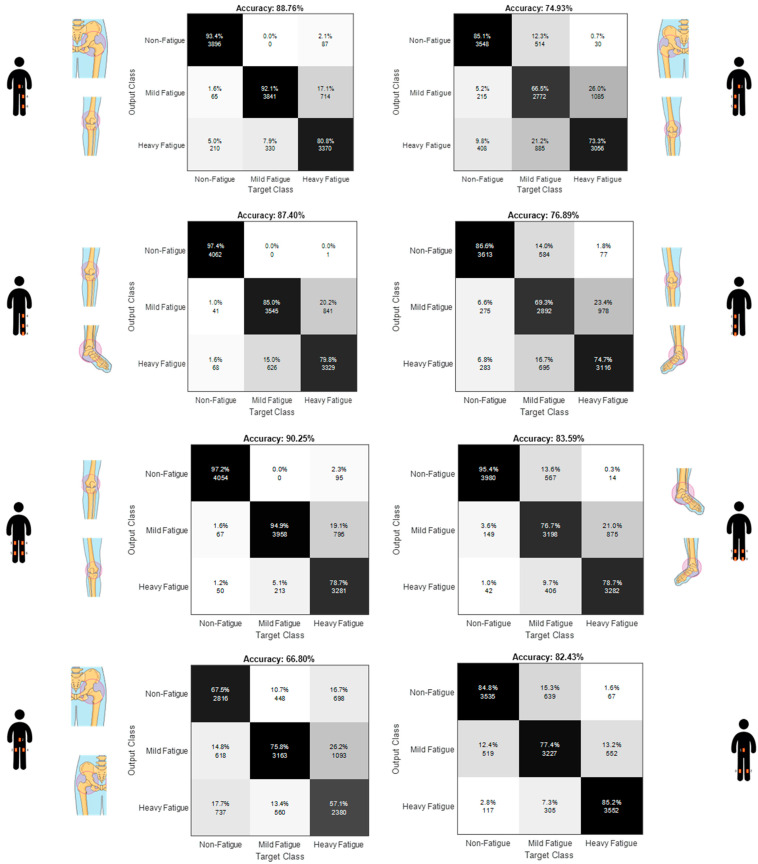
Aggregate confusion matrices with all subjects, 3+ IMUs configurations, up to two joints. Joints present in each configurations are shown.

**Figure A4:**
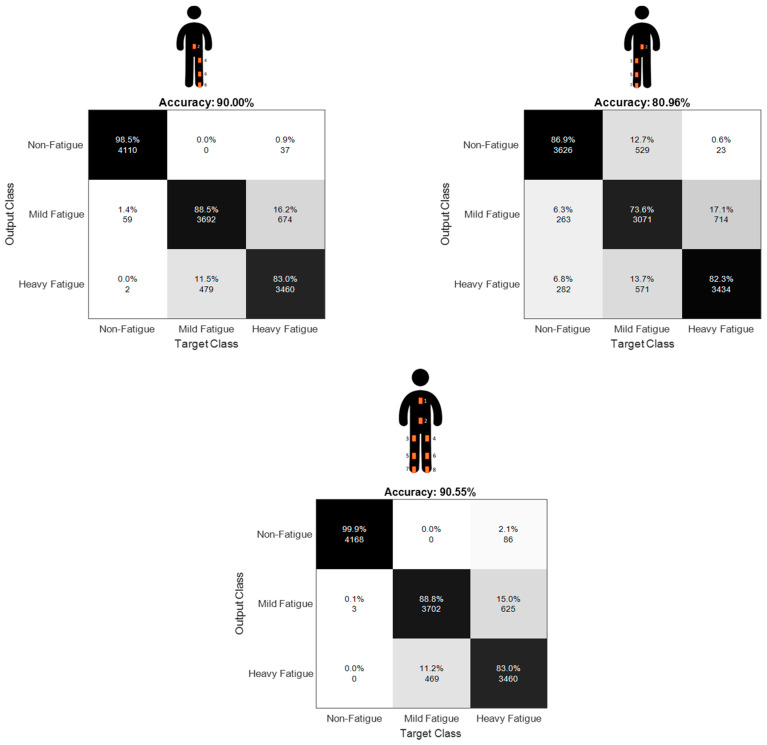
Aggregate confusion matrices with all subjects, whole lower limbs and full body configurations.

Full assessment of the different sensor configurations for each category is shown by means of confusion matrices in Appendix A, respectively in Figure A1 (minimally intrusive configuration), Figure A2 (quasi-minimally intrusive configuration), Figure A3 (3+ IMUs configuration) and Figure A4 (full limbs and whole body configurations). Left limb sensors outperform right limb sensors in the minimally intrusive configurations, although the difference at the tibia segment (best location) is almost negligible. Configurations including at least one knee joint perform better than configurations without a knee joint, both in the quasi-minimally intrusive and 3+ IMUs configurations. Using our experimental paradigm, we find that increasing the number of sensors generally increases performance of the machine learning classification algorithm. However, adding one sensor could also slightly decrease accuracy of the classifier, although this was not observed when the additional sensor also resulted in one additional joint angle.

Table 6 presents the five higher ranked features across participants in the leave-one-subject-out cross validation approach. The most recurring feature is the STD of the tibial pitch angular velocity. Each configuration that includes joint angles presents at least one biomechanical feature derived from a joint angle. Each configuration shows at least one biomechanical and one statistical feature in the best five features. Features from the left limb are predominant in configurations with both limbs present. Spatiotemporal, symmetry and shock attenuation features are not present as best features in any configuration.

## 4. Discussion

The purpose of this study was to assess the performance of a machine learning algorithm to detect fatigue during a prolonged outdoor run using single or multiple IMUs. We assessed the detection accuracy of selected IMU configurations of interest and the trade-off between higher fatigue classification accuracy and sensor reduction.

### 4.1. Sensor Location Optimization

We demonstrated in this study how various minimal sensor setups can be able to detect fatigue at satisfying levels when sensor location is chosen wisely. We obtained an accuracy above 76% using a random forest algorithm with only one IMU sensor and 12 features. This is in line with previous studies performed to detect fatigue in running and work tasks. An AUC-ROC of 0.68 [25] and an accuracy of 75% [19] were already found in running using a single IMU sensor on the tibia, although with different fatiguing protocols and device types. While single IMU configurations present a clear advantage of a low intrusiveness, we used a structured approach to evaluate fatigue detection performance of IMU setups up to eight sensor locations. We observed that increasing the number of IMU locations from one to two leads to an improvement in accuracy up to 87%, while increasing the number of IMU locations to four leads to an improvement in accuracy up to 90% that remains at the same level when increasing the number of IMU locations to eight.

Fatigue detection accuracy was highest at the tibias, both in minimally intrusive and more intrusive configurations. We expected that the tibias would generate the best fatigue detection performance due to the documented changes in peak tibial acceleration due to running-induced fatigue [13,14,15]. However, the most recurring features with highest importance in the best configurations were linked to the variation of tibial pitch angular velocity in the sagittal plane and acceleration magnitude. Statistical features that are indicative of gait variability were also among the features with highest importance.

IMU configurations with an increasing number of joint angles resulted in an increase of accuracy. For example, the left thigh and foot in minimally intrusive configurations resulted in a low level of accuracy (respectively 60% and 59%). Still, when coupled to the left tibia sensor in a three IMUs configuration they resulted in an accuracy of 87%. Knee and ankle joints resulted in considerably higher accuracies in the quasi-minimally intrusive configurations compared to minimally intrusive configurations without joint angles. However, increasing the number of joint angles from one to two, two to three and three to six resulted in minimal increases in accuracy. This indicates that knee and ankle joints are more suited than hips to detect fatigue in outdoor runs using IMUs. Gholami et al. obtained opposite results using textile wearable sensors to detect fatigue in running, with the hip being the most reliable sensor location and the knee and ankle being less reliable [18]. However, wearable sensors used in the study measured biomechanical parameters only in the sagittal plane. The trade-off observed between number of sensors and detection accuracy in the present study suggests a sensor setup including only one joint angle, preferably the left or right knee.

IMUs placed on the left lower limb generally resulted in higher fatigue detection accuracy than the right lower limb (e.g., full left lower limb accuracy = 0.900, full right lower limb accuracy = 0.809). Since only recreational runners were included, it was not possible to reliably estimate their dominant leg in running. However, a change of direction was included halfway through the runs in the running protocol to eliminate the effect of running direction on the biomechanics of the left and right leg. Leg dominance had already been found not to have effects on kinematic differences due to fatigue in running [26]. Further studies are needed to confirm whether the non-dominant leg is better suited for sensor placement when detecting fatigue.

### 4.2. Machine Learning and Biomechanics

Machine learning has been successfully used in plenty of biomedical applications in recent years. While the amount of publications involving machine learning increases almost every year since the early 2000s [27], the field of biomechanics is still anchored to salient features such as peak tibial accelerations and peak joint angles. Traditional biomechanical parameters derived by IMU measurements have the advantage of being highly interpretable. However, they might not fully capture some underlying mechanisms such as gait variability due to fatigue. While statistical variables might not show significant differences, they still relate to an underlying running gait variability that is expected to increase with fatigue [28]. We observed in this study that a machine learning approach to detect fatigue in running has benefited from both statistical and biomechanical parameters, as already shown in studies performed in work scenarios [29].

Traditional biomechanics focus predominantly on group level averages of salient variables. However, previous studies have shown that changes in specific variables are extremely subject-dependent [8,9,14]. Subject-specific characteristics (e.g., running experience, body morphology, gender, speed) can influence running biomechanics, often not allowing drawing conclusions at a subject level as well as at group level. Machine learning applications in biomechanics have the potential to fill this gap. By applying leave-one-subject-out cross validation, machine learning algorithms can make predictions on subjects that were never observed before. This technique can help identifying biomechanical features that best describe the predicted outcome (e.g., fatigue) on different subjects, improving generalization of the prediction on new subjects.

### 4.3. Toward Real-World Applications

The deployment of a large scale IMU-based fatigue detection device remains a challenge. The current study aimed to add knowledge in the translation of biomechanical changes due to fatigue into a real-world application. We introduced the use of a moving average in detection of fatigue in running using IMUs. Such technique has two main implications. First, the algorithm would not give live feedback, but a delay of the duration of the moving average (e.g., time to complete one athletic track full lap) would be present. Second, the classification algorithm could be specific for a run on an athletic track. However, running on a track is a very popular option for runners. Further studies should be performed to apply this classifier to other running scenarios (i.e., different durations, intensities, surfaces), although taking into account that IMUs placement and skin displacements could affect the results.

A satisfying level of fatigue detection accuracy for real-world applications is difficult to determine. Every runner is different, and so are runners’ expectations and interactions with a fatigue detection device. While recreational runners could be satisfied with an average fatigue detection score at the end of a run, elite runners would likely require more detailed information about fatigue progression throughout a training session. While a minimal threshold for fatigue detection accuracy cannot be universally established, it is important to remark how a balance between sensitivity and specificity should be pursued. In fact, a low sensitivity would result in a system that cannot be trusted by the runner, while a low specificity would result in many false alarms that can mine the willingness of the runner to use the device.

### 4.4. Limitations

A first limitation of this study was the definition of fatigue levels. RPE scales have been widely used as a measuring tool for perception of fatigue, since RPE has been already found to be a reliable surrogate for exercise intensity [30], but they do not represent a gold standard for measurement of physical fatigue. During the last, heavy fatigue condition of our experimental protocol we observed on average a decrease of RPE, although very subject-dependent. This was probably due to a sudden decrease in speed from the previous fatiguing protocol and could have encumbered the classifier ability to distinguish between a mild and heavy fatigue condition. While a change in running intensity could impact perception of fatigue, performance fatigability might still be increasing at a muscle level.

A second limitation of this study is related to the amount of IMU combinations taken into account. It would have been impractical to analyze all 255 combinations deriving from the eight IMU locations chosen in the present study. However, we believe that our set of assessed configurations includes the ones of highest relevance in real-world applications. Configurations with five to seven IMU locations were excluded because they were not expected to differ significantly from the whole body configuration with eight IMUs, while less intrusive sensor setups were mostly reduced to IMU configurations including at least one joint angle.

A third limitation regards the relatively small sample size of eight subjects that participated in the study. Although conducting a similar study with a larger population would present benefits with respect to generalization of the results and would consent to draw appropriate statistical conclusions, the large number of strides generated per subject and the leave-one-subject-out cross-validation approach allowed our classification algorithm to generalize well to unseen data.

### 4.5. Future Research

Objective assessment of fatigue would have direct benefits for a runner. We suggest validating IMU-based techniques against gold standards in the detection of physical fatigue such as electromyography (EMG) and maximal oxygen consumption (VO2 max). Furthermore, performing IMU-based fatigue detection studies with larger, less homogeneous populations could allow the application of deep learning techniques and help generalizing a fatigue detection algorithm more widely.

## 5. Conclusions

We assessed the optimal combination of IMU locations at the lower limbs and trunk to detect fatigue levels in an outdoor run. Machine learning techniques allowed to learn from IMU-derived biomechanical and statistical parameters and detect fatigue in prolonged running activities with increasingly higher accuracy from a single IMU location up to eight locations. The tibias and the knees are respectively the IMU locations and joint angles resulting in higher fatigue detection accuracy. We recommend using IMU configurations consisting of one to four sensors that include at least one of the tibias for fatigue detection in young recreational runners.

## Figures and Tables

**Table 1:** Runners characteristics.

Subject	Age (Years)	Body Mass (kg)	Height (cm)	Speed (km/h)	Running Experience (Years)	Sex
S001	25	69	182	13.0	5	M
S002	24	55	164	10.5	3	F
S003	23	69	167	9.1	9	F
S004	24	64	168	9.6	7.5	F
S005	25	78	174	9.4	2	F
S006	26	77	187	11.6	1.5	M
S007	23	75	169	9.9	1.5	F
S008	24	82	188	11.6	6	M
Mean(±1STD)	24.3 ± 1.0	71.1 ± 8.8	174.8 ± 9.5	10.6 ± 1.4	4.4 ± 2.8	-

**Table 2:** Features extracted for each body location. Features were extracted from acceleration and angular velocities of eight body segments (top) and from the angles of six joints (bottom). Additionally, shock attenuations were computed between the left tibia and pelvis, and between the right tibia and pelvis.

Body Segments																							
Left Foot			Right Foot			Left Tibia			Right Tibia			Left Hip			Right Hip			Pelvis			Sternum		
**Biomechanical features**																							
Peak acceleration			Peak acceleration			Peak acceleration			Peak acceleration			Peak acceleration			Peak acceleration			Peak acceleration			Peak acceleration		
Peak ang. Vel ^1^			Peak ang. vel			Peak ang. vel			Peak ang. vel			Peak ang. vel			Peak ang. vel			Peak ang. vel			Peak ang. vel		
						Shock attenuation (with pelvis)			Shock attenuation (with pelvis)														
**Statistical features**																							
Mean acceleration			Mean acceleration			Mean acceleration			Mean acceleration			Mean acceleration			Mean acceleration			Mean acceleration			Mean acceleration		
STD acceleration			STD acceleration			STD acceleration			STD acceleration			STD acceleration			STD acceleration			STD acceleration			STD acceleration		
IQR acceleration			IQR acceleration			IQR acceleration			IQR acceleration			IQR acceleration			IQR acceleration			IQR acceleration			IQR acceleration		
Skew. acceleration			Skew. acceleration			Skew. acceleration			Skew. acceleration			Skew. acceleration			Skew. acceleration			Skew. acceleration			Skew. acceleration		
Kurt. acceleration			Kurt. acceleration			Kurt. acceleration			Kurt. acceleration			Kurt. acceleration			Kurt. acceleration			Kurt. acceleration			Kurt. acceleration		
Mean ang. vel.			Mean ang. vel.			Mean ang. vel.			Mean ang. vel.			Mean ang. vel.			Mean ang. vel.			Mean ang. vel.			Mean ang. vel.		
STD ang. vel.			STD ang. vel.			STD ang. vel.			STD ang. vel.			STD ang. vel.			STD ang. vel.			STD ang. vel.			STD ang. vel.		
IQR ang. vel.			IQR ang. vel.			IQR ang. vel.			IQR ang. vel.			IQR ang. vel.			IQR ang. vel.			IQR ang. vel.			IQR ang. vel.		
Skew. ang. vel.			Skew. ang. vel.			Skew. ang. vel.			Skew. ang. vel.			Skew. ang. vel.			Skew. ang. vel.			Skew. ang. vel.			Skew. ang. vel.		
Kurt. ang. vel.			Kurt. ang. vel.			Kurt. ang. vel.			Kurt. ang. vel.			Kurt. ang. vel.			Kurt. ang. vel.			Kurt. ang. vel.			Kurt. ang. vel.		
**Joint angles**																							
**Left ankle**				**Right ankle**				**Left knee**				**Right knee**				**Left hip**				**Right hip**			
**Biomechanical features**																							
IC ^1^ (peak)				IC (peak)				IC (peak)				IC (peak)				IC (peak)				IC (peak)			
Mid Stance (peak)				Mid Stance (peak)				Mid Stance (peak)				Mid Stance (peak)				Toe off (peak)				Toe off (peak)			
Toe off (peak)				Toe off (peak)				Toe off (peak)				Toe off (peak)				Mid Swing (peak)				Mid Swing (peak)			
Left-right difference								Mid Swing (peak)				Mid Swing (peak)				Left-right difference							
								End Swing (peak)				End Swing (peak)											
								Left-right difference															
**Statistical features**																							
Mean				Mean				Mean				Mean				Mean				Mean			
STD				STD				STD				STD				STD				STD			
IQR				IQR				IQR				IQR				IQR				IQR			
Skewness				Skewness				Skewness				Skewness				Skewness				Skewness			
Kurtosis				Kurtosis				Kurtosis				Kurtosis				Kurtosis				Kurtosis			

^1^ ang. vel. = pitch angular velocity; IQR = inter-quartile range; STD = standard deviation; Skew = skewness; Kurt = kurtosis; IC = initial contact.

**Table 3:** Possible sensor combinations with a total number of IMUs (n) = 8.

Number of IMUs in a Configuration (*r*)	$\mathrm{Number}\;\mathrm{of}\;\mathrm{Combinations}\;(C)\;C\left(n,r\right)\;=\;\frac{n!}{\left(r!\left(n-r\right)!\right)}\;,\;n\;=\;8$
1	8
2	28
3	56
4	70
5	56
6	28
7	8
8	1
Total	255

**Table 4:** Selected sensor combinations and number of lower limb joints per configuration.

Minimally Intrusive	Quasi-Minimally Intrusive	3 + IMUs	Whole Body
L1 (0 joints)	L2 L3 (1 joint)	L2 L5 L6 (0 joints)	L1 L2 L3 L4 L5 L6 L7 L8 (6 joints)
L2 (0 joints)	L2 L4 (1 joint)	L2 L3 L4 (2 joints)	
L3 (0 joints)	L3 L5 (1 joint)	L2 L3 L5 (2 joints)	
L4 (0 joints)	L4 L6 (1 joint)	L2 L4 L6 (2 joints)	
L5 (0 joints)	L5 L7 (1 joint)	L3 L5 L7 (2 joints)	
L6 (0 joints)	L6 L8 (1 joint)	L4 L6 L8 (2 joints)	
L7 (0 joints)	L3 L4 (0 joints)	L3 L4 L5 L6 (2 joints)	
L8 (0 joints)	L5 L6 (0 joints)	L5 L6 L7 L8 (2 joints)	
	L7 L8 (0 joints)	L2 L3 L5 L7 (3 joints)	
		L2 L4 L6 L8 (3 joints)	

**Table 5:** Performance metrics for the three fatigue classes (Mean ± STD). Mean calculated over all datapoints from all left-out-subjects.

Category	Best IMU Locations	Level	Sensitivity	Specificity	Precision	F1 Score
Minimally intrusive	L6 (left tibia)	No fatigue	0.774 ± 0.318	0.895 ± 0.151	0.798 ± 0.283	0.774 ± 0.285
		Mild fatigue	0.645 ± 0.387	0.855 ± 0.188	0.703 ± 0.342	0.637 ± 0.343
		Heavy fatigue	0.865 ± 0.179	0.893 ± 0.140	0.836 ± 0.172	0.834 ± 0.146
Quasi-minimally intrusive	L4 L6 (left thigh, left tibia)	No fatigue	0.949 ± 0.105	0.988 ± 0.035	0.979 ± 0.059	0.960 ± 0.063
		Mild fatigue	0.898 ± 0.172	0.879 ± 0.118	0.809 ± 0.166	0.840 ± 0.144
		Heavy fatigue	0.748 ± 0.234	0.931 ± 0.089	0.852 ± 0.168	0.783 ± 0.184
3+ IMUs	L3 L4 L5 L6 (right thigh, left thigh, right tibia, left tibia)	No fatigue	0.973 ± 0.050	0.986 ± 0.039	0.978 ± 0.064	0.974 ± 0.039
		Mild fatigue	0.949 ± 0.069	0.894 ± 0.109	0.843 ± 0.147	0.885 ± 0.085
		Heavy fatigue	0.776 ± 0.273	0.969 ± 0.033	0.931 ± 0.058	0.819 ± 0.188
Whole body	L1 L2 L3 L4 L5 L6 L7 L8	No fatigue	0.999 ± 0.002	0.987 ± 0.035	0.979 ± 0.059	0.988 ± 0.032
		Mild fatigue	0.885 ± 0.116	0.927 ± 0.099	0.881 ± 0.155	0.875 ± 0.113
		Heavy fatigue	0.830 ± 0.192	0.942 ± 0.058	0.879 ± 0.126	0.846 ± 0.144

**Table 6:** Best features in each configuration. In bold features extracted from joint angles.

Feature Rank	Left Tibia	Left Tibia + Left Thigh	Right Thigh + Left Thigh + Right Tibia + Left Tibia	All IMU Locations
#1	STD acceleration	***Toe off minimum knee angle***	STD angular velocity left tibia	STD angular velocity left tibia
#2	STD angular velocity	STD angular velocity tibia	***Mid stance maximum left knee angle***	***Skewness left ankle angle***
#3	Mean angular velocity	***STD knee angle***	STD angular velocity right tibia	***Mid stance maximum left knee angle***
#4	Peak angular velocity	Peak angular velocity tibia	STD acceleration left tibia	STD acceleration left tibia
#5	IQR angular velocity	Mean angular velocity tibia	Peak angular velocity right tibia	***Mid stance maximum left ankle angle***

## Data Availability

The data presented in this study are openly available in 4TU. Research. Data at [https://doi.org/10.4121/14307743], last accessed 14 May 2021.
